# Past, present, and future strategies for detecting and quantifying circular RNA variants

**DOI:** 10.1111/febs.70012

**Published:** 2025-02-11

**Authors:** He Lin, Vanessa M. Conn, Simon J. Conn

**Affiliations:** ^1^ Flinders Health and Medical Research Institute, College of Medicine & Public Health Flinders University Adelaide Australia

**Keywords:** alternative splicing, circRNAs, circular RNAs, long‐read sequencing, next‐generation sequencing

## Abstract

Circular RNAs (circRNAs) are a family of covalently closed RNA transcripts ubiquitous across the eukaryotic kingdom. CircRNAs are generated by a class of alternative splicing called backsplicing, with the resultant circularization of a part of parental RNA producing the characteristic backsplice junction (BSJ). Because of the noncontiguous sequence of the BSJ with respect to the DNA genome, circRNAs remained hidden in plain sight through over a decade of RNA next‐generation sequencing, yet over 3 million unique circRNA transcripts have been illuminated in the past decade alone. CircRNAs are expressed in a cell type‐specific manner, are highly stable, with many examples of circRNAs being evolutionarily conserved and/or functional in specific contexts. However, circRNAs can be very lowly expressed and predictions of the circRNA context from BSJ‐spanning reads alone can confound extrapolation of the exact sequence composition of the circRNA transcript. For these reasons, specific and ultrasensitive detection, combined with enrichment, bespoke bioinformatics pipelines and, more recently, long‐read, highly processive sequencing is becoming critical for complete characterization of all circRNA variants. Concomitantly, the need for targeted detection and quantification of specific circRNAs has sparked numerous laboratory‐based and commercial approaches to visualize circRNAs in cells and quantify them in biological samples, including biospecimens. This review focuses on advancements in the detection and quantification of circRNAs, with a particular focus on recent next‐generation sequencing approaches to bolster detection of circRNA variants and accurately normalize between sequencing libraries.

## Graphical abstract

Originally considered artifact of RNA splicing, over 3 million circular RNAs have been found in humans alone through a combination of ultrasensitive detection methods and bespoke bioinformatics tools. This review covers past, present, and future strategies for detecting and quantifying circular RNAs and how they are illuminating novel circular RNA variants. Increasing reports demonstrating functional roles for circular RNAs in human health underscore the need for robust and comprehensive detection.

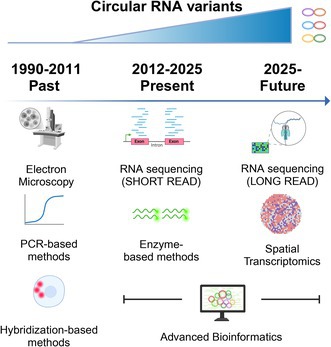

**Abbreviations**
AS: alternative splicing
bp: base pairs
BSJ: backsplice junction
Capture‐seq: capture sequencing
circRNA: circular RNA
ciRNA: intron‐only circRNA
CRISPR: clustered regularly interspaced short palindromic repeats
ddPCR: droplet digital PCR
DSN: duplex‐specific nuclease
EcircRNA: exon‐only circRNA
EIciRNA: exon‐intron circRNA
LAMP: loop‐mediated isothermal amplification
LNA: locked nucleic acid
LNA: locked nucleic acids
ME: microexon
mRNA: messenger RNA
NGS: next‐generation sequencing
nt: nucleotides
PCR: polymerase chain reaction
qRT‐PCR: quantitative real‐time reverse transcription PCR
RCA: rolling circle amplification
RI: retained introns
RPAD: RNase R treatment, polyadenylation and poly(A)+ RNA depletion
RT‐PCR: reverse transcription PCR
SA: splicing acceptor
SD: splicing donor
SLP: stem‐loop primer
SynCRS: synthetic circular RNA spike‐ins

## Introduction

**Fig. 1:**
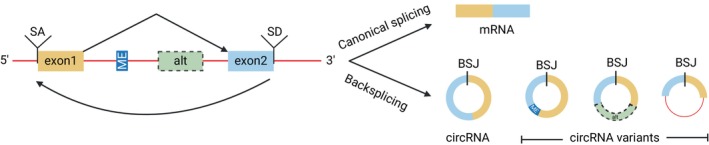
The formation of linear mRNAs and circRNA through canonical splicing and backsplicing, respectively. Backsplicing occurs from the 3′ to 5′ direction, a downstream splicing donor (SD) site is ligated to an upstream splicing acceptor (SA) site, forming a unique junction called the backsplice junction (BSJ). Multiple types of circRNAs are generated via backsplicing sharing the same BSJ, including microexon (ME), alternative exon (alt, in green), and intron (red line). Created with BioRender.com.

CircRNAs are formed by noncanonical alternative splicing (AS) events, collectively known as backsplicing. Unlike pre‐mRNAs that undergo splicing largely from the 5′ to 3′ direction, backsplicing involves the ligation of a splicing donor site to an upstream splicing acceptor site of the same pre‐mRNA transcript [1]. This produces a unique junction site, called a backsplice junction (BSJ), whose preceding and succeeding sequence is derived from the parental mRNA, but which is now present in a noncontiguous order (Fig. 1). Backsplicing is guided by the spliceosome, in concert with *cis*‐acting and *trans*‐acting factors, including the presence of inverted repeat elements in flanking introns and a range of RNA binding proteins and splicing factors, respectively [2]. Most circRNAs contain one or more exons (EcircRNA), while there is an expanding portfolio of circRNAs comprising both exons and introns (EIciRNA), introns alone (ciRNA), or intergenic sequences [1]. CiRNAs are distinct from other circRNAs, which are formed via backsplicing. CiRNAs are specifically generated from intronic regions of pre‐mRNA and do not involve exon–exon backsplicing.

As the definitive feature of the circRNA, the BSJ is exploited in the identification and quantification of the circRNA from high‐throughput RNA sequencing, design of oligonucleotide primers, and for RNA interference strategies to distinguish it from its cognate linear RNA. Bioinformatics pipelines exploit BSJ‐spanning reads and require a minimum number of bases on either side of the BSJ to accurately map the beginning and endpoint of each circRNA [3]. The vast majority of NGS methods utilizes short‐read sequencing (75–300 nucleotides) to identify the circRNA, with paired‐end sequencing being preferred as it provides more sequence context. CircRNAs are generally present at much lower abundance than mRNAs due to backsplicing being less efficient than canonical splicing [4]. However, even with strategies to enrich for circRNAs, typically < 10% of NGS reads correspond to circRNAs [5, 6]. Furthermore, with bioinformatics tools restricted to defining the genomic coordinates of the beginning and end of the specific circRNA only, the composition of the entire circRNA (hereafter referred to as the circRNA body) can only be inferred from short‐read NGS. These factors complicate the detection of circRNA variants, which exhibit the same BSJ yet contain alternate sequences in the circRNA body, including unique whole exons, partial exons and microexons [7, 8, 9], intergenic regions [10], and introns [11] (Fig. 1). Therefore, it is critical that, following RNA‐seq, the internal structure of the circRNA is defined to ensure that subsequent circRNA quantification is specific for the targeted circRNA variant(s).

As an indication of the depth of circRNA variants, the FL‐circAS database, which integrates nanopore long‐read sequencing data, identified over 1.8 million circRNA variants from only 884 636 BSJs in human (isoform ratio of ~ 2.1), while for mouse, there is less variation detected with 135 617 full‐length circRNA variants from 115 173 BSJs (isoform ratio of ~ 1.2) [12]. Additional variants arise from circRNAs containing microexons (ME‐circRNAs), which account for ~ 3.5% of all circRNAs. ME‐circRNAs possess microexons at their BSJ as either completely novel circRNAs, or as circRNA variants with over 600 of these having a cognate circRNA possessing the same BSJ but lacking the microexon in glioblastoma tumor tissue [7]. The inclusion of a unique whole exon is commonly observed in alternatively spliced circRNAs. One example is the human *TIMMDC1* gene, which produces two circRNA variants, *circTIMMDC1(2,4)* and *circTIMMDC1(2,3,4)*, which share the same BSJ between the end of exon 4 and the beginning of exon 2 but differ in the exclusion or inclusion of exon 3 [13]. Moreover, circRNAs can incorporate retained introns (RI) that are typically spliced out during canonical mRNA splicing. For instance, a circRNA variant from the human *REXO4* gene incorporates RI in its structure, circREXO4(2,RI,3) [14].

Beyond their expression in tissues and cell lines, circRNAs are exported from cells in extraceullar vesicles and have been identified in a range of biospecimens, including blood and blood products (serum, plasma, and platelets) and bodily fluids (saliva, feces, and urine) (reviewed in Ref. [15]). The hyperstability of circRNAs combined with their disease‐specific expression profile, particularly in cancer tissue, make circRNAs a very good candidate as a disease biomarker. As a result, alternate strategies for the identification and sensitive quantification of circRNA variants are now in high demand. This review will summarize the salient aspects of circRNA from enrichment to techniques to detect and profile circRNAs *de novo* (NGS) and to detect and quantify specific circRNAs with and without amplification. It will also highlight a recent methodological advance, which overcomes many of the challenges for circRNA variant profiling.

## CircRNA enrichment strategies

CircRNAs account for < 0.01% of the total cellular RNA pool, compared with ribosomal RNA (80–90%), transfer RNAs (10–15%) and mRNA (2–3%); therefore, enriching for circRNAs before detection may be advantageous or necessary depending on the methodology employed, or the specific scientific aim [16].

**Fig. 2:**
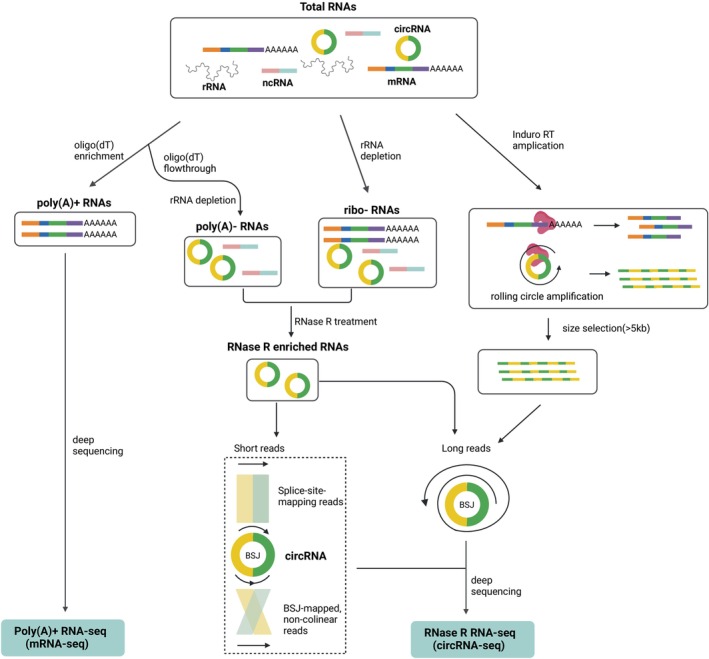
CircRNA enrichment strategies preceding next‐generation sequencing. As distinct from mRNA‐seq where polyadenylated (poly(A)+ RNA) is enriched, circRNAs are enriched from total RNA is subjected to linear RNA removal via ribosomal RNA (rRNA) depletion or depletion of poly(A)+ RNA (poly(A)− pool) followed by RNase R treatment. Induro‐RT‐mediated circRNA enrichment is achieved without RNase R treatment through rolling circle amplification, followed by bead purification to enrich long concatemeric cDNA produced from circRNAs from shorter linear RNAs. Created with BioRender.com.

The enrichment of circRNAs is predominantly achieved through digestion with ribonuclease R (RNase R), a 3′–5′ exonuclease that preferentially degrades unstructured linear RNAs while preserving the vast majority of circRNAs due to their lack of termini (Fig. 2). However, some linear RNAs containing highly structured regions, such as G‐quadruplexes, or lack 3′ overhangs and are recalcitrant to RNase R digestion [17]. While these remnant linear RNAs can absorb sequencing depth, there are further complications when *trans*‐splicing occurs (splicing between two separate linear RNAs) to produce an apparent BSJ resulting in false‐positive identification of circRNAs [18, 19]. Improvements in this enrichment strategy have substituted potassium chloride with lithium chloride in the digestion buffer to reduce G‐quadruplexes and enhance the degradation of structured linear RNAs [17]. False negatives may also arise from star activity of the RNase R, particularly for larger circRNAs, which undergo scission more frequently than smaller circRNAs and when incubation times are extended [20]. Additionally, variations in RNase R concentration or discrepancies between different batches can significantly influence the rates of both false positives and false negatives [21]. These considerations highlight the necessity for standardization of experimental conditions and rigorous validation of methodologies to ensure robust and reproducible circRNA enrichment and characterization.

Situations where RNase R treatment would not be recommended include when calculating the circular‐to‐linear RNA ratio by Northern blotting or qRT‐PCR, unless a mock treatment control is performed side‐by‐side to limit experimental bias. Moreover, abundant circRNAs can still be detected in total RNA samples that have not been subjected to RNase R treatment. Additionally, in the context of deep next‐generation sequencing, it may be more beneficial to employ ribosomal RNA (rRNA) removal rather than RNase R treatment, especially when utilizing degraded RNA or when the goal is to capture a comprehensive RNA profile and optimize sequencing depth. In some instances, enrichment strategies may be unnecessary, particularly when using targeted assays such as qRT‐PCR, where enrichment is not typically required and could complicate the detection or quantification of circRNAs with a linear housekeeping transcript.

RNase R Treatment, Polyadenylation, and Poly(A)+ RNA Depletion (RPAD) is another enrichment method designed for the isolation of circRNAs. This technique employs a sequential approach involving RNase R treatment to selectively degrade linear RNAs, followed by polyadenylation of residual linear RNAs and depleting poly (A) RNAs [22]. RPAD may negatively bias those circRNAs containing polyA‐stretches such as CDR1as [14] and is not so widely employed.

Capture sequencing (Capture‐seq) is a powerful, poly(A)‐independent method designed to selectively enrich specific genomic regions for detailed analysis of particular genes or loci [23]. This approach involves hybridizing RNA with a panel of bespoke, biotinylated probes that bind to the desired sequences, including circRNA BSJs, followed by capturing these bound target RNAs using streptavidin‐coated beads. This effectively enriches the target sequences while minimizing carryover of off‐target RNA without need for enzymatic digestion. This method was employed to develop MiOncoCirc, a comprehensive database that identifies circRNAs across 2000 cancer samples comprising a range of cancers [23].

As circular and linear RNAs migrate distinctly through gel matrices [24], another potential strategy for circRNA enrichment, yet to be widely applied, is the use of two‐dimensional polyacrylamide gel electrophoresis where the circular and linear RNAs migrate as distinct parallel diagonals, through the gel [25, 26]. A commercial version of this strategy was achieved via multicapillary electrophoresis, to achieve separation of a single linear and circular RNA species [27]. Whether this would be adaptable to achieve separation of a complex mixture of RNA molecules remains to be established.

## *De novo* identification of circRNAs by RNA‐seq

Purified and/or enriched RNA containing circRNAs are subjected to library construction for short‐read or long‐read NGS analysis. As circRNAs lack termini, random primers are required for first‐strand complementary DNA (cDNA) synthesis to detect the greatest number of circRNAs.

Bioinformatic pipelines to predict circRNAs relies on sequencing reads overlapping the BSJ, which complicates the ability to dissect the entire structure of circRNAs when using short‐read NGS (typically 50–300 nucleotide read lengths). For this reason, paired‐end (PE) sequencing offers distinct advantages over single end sequencing [8, 28]. Because the average exon is about 150 nucleotides [29], and most commonly circRNAs contain two or three exons [30], longer read sequencing platforms, including Oxford Nanopore Technologies (ONT) and Pacific Biosciences (PacBio) platforms, are more efficient for mapping the entire circRNA and detecting variants. Long‐read sequencing approaches, including isoCirc [5] and circFL [6], which exploit rolling circle amplification using random primers and circRNA panel long‐read sequencing (circPanel‐LRS) using circRNA‐specific primers [14], can produce reads of thousands to tens of thousands of bases in length. This provides end‐to‐end circRNA context and has revealed a large number of splicing events exist within the circRNA body [6, 7, 12, 14]. The FLcirc‐AS database, consolidating long‐read sequencing data for circRNAs, has found over 1.8 million circRNA variants [12].

Common bioinformatics tools such as CIRI2 [31], find_circ [32], and CIRCexplorer2 [33] all aim to identify these BSJs from short‐read RNA sequencing but differ in their strategies, sensitivity, and unsurprisingly the profile of circRNAs, which are outputted. Bespoke tools tailored for interrogating other sequencing approaches are also available, including CIRI‐long for long‐read sequencing [34] and CIRI‐Deep for spatial transcriptomics/single‐cell sequencing [35]. While beyond the scope of this review, a contemporary benchmarking study on bioinformatic tools for circRNA identification has been published and is useful for researchers seeking greater insight into this critical aspect [3].

## Induro‐RT‐mediated circular RNA sequencing (IMCR‐seq)

Recently, a novel method was developed to address many issues previously highlighted in the detection of circular RNA variants called Indro‐RT‐mediated circRNA‐sequencing (IMCR‐seq) [36]. IMCR‐seq uses a group II intron reverse transcriptase with strong strand displacement activity to enhance rolling circle reverse transcription even in the presence of strong secondary structure, to produce very long cDNA transcripts, which are purified form shorter transcripts (linear RNAs) by bead selection (Fig. 2). This approach produces concatameric sequences, even from short circRNAs and, in combination with long‐read sequencing (PacBio or Oxford Nanopore are commonly used platforms) and CIRI‐long bioinformatics pipeline, achieved 6–1000 times greater circRNA variant detection in human samples compared with other methods [36]. Furthermore, boosting the versatility of this approach, libraries could be generated from as little as 10 ng of RNA, with serum profiling of circRNAs from lung cancer patients identifying enriched circRNAs, including a unique intron‐containing circRNA variant from RAB3IP [36]. The vastness of these types of circRNA variants is only now being appreciated, opening the door for greater functional investigation of these RNA transcripts. For quantitative comparison of circRNAs between sequencing libraries, synthetic circular RNA spike‐ins (SynCRS) are strongly encouraged as a normalization method for all future circRNA‐seq, especially where circRNA concentrations in biospecimens are critical for ascribing clinical relevance [37].

## Laboratory methods for validation and quantification of circRNAs

**Fig. 3:**
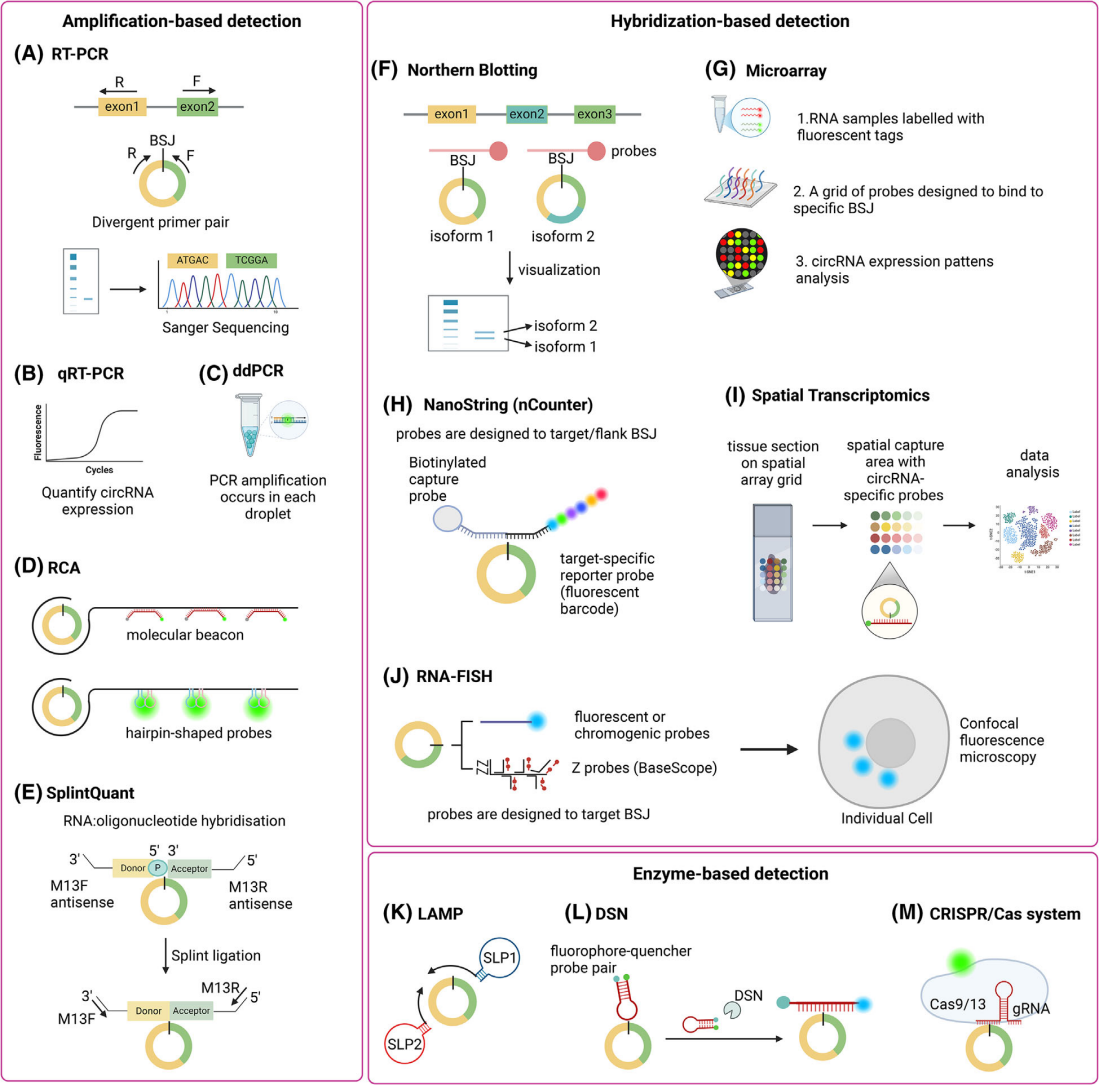
CircRNA detection methods. PCR‐based detection methods for circRNAs including (A) Reverse transcription PCR (RT‐PCR), (B) quantitative, real‐time RT‐PCR (qRT‐PCR), (C) digital droplet PCR (ddPCR), (D) Rolling circle amplification (RCA), and (E) SplintQuant using PBCV‐1 DNA ligase all employing divergent primer pairs for amplification across the BSJ. Hybridization‐based detection with probes targeting the BSJ or body of the circRNA including (F) Northern blotting to determine size and quantify circRNA isoforms, (G) Microarray, (H) NanoString nCounter assay using unique fluorophore barcoding and (I) Spatial transcriptomics using circRNA‐specific probes and (J) RNA fluorescence *in situ* hybridization (FISH), (using fluorescent or chromogenic probes, or BaseScope technology) to localize circRNAs intracellularly. Hybridization‐based detection methods. Enzyme‐based detection methods including (K) Loop‐mediated isothermal amplification (LAMP) with stem‐loop primers (SLP), (L) duplex‐specific nuclease (DSN) and (M) deactivated CRISPR‐Cas detection using guide RNA targeting the circRNA BSJ. Created with Biorender.com.

Once candidate circRNAs have been identified it is critical to validate these with orthogonal methods, with RT‐PCR and Northern blotting the most commonly employed techniques. Following validations, quantification of the circRNA abundance in purified RNA and intracellular visualization are considered best practice for research purposes [20], with a range of methods developed (Fig. 3).

## Amplification‐based circRNA detection

### Reverse transcriptase polymerase chain reaction (RT‐PCR)

RT‐PCR is one of the foundational techniques for detecting circRNAs, often followed by agarose gel electrophoresis and Sanger sequencing to validate the expected size(s) and sequence. In this method, circRNAs are detected using specific oligonucleotides flanking the circRNA BSJ (Fig. 3A). This approach to primer design may lead to the amplification of more than one circRNA isoform if the BSJ harbors unique sequences, including microexons. Furthermore, multiple distinct circRNA variants sharing the same BSJ (Fig. 1) could be incorrectly quantified as a single species if the oligonucleotide primers are complementary to regions that are shared among these circRNAs. This potential confounding can be partially mitigated by designing one of the oligonucleotides to span the BSJ. However, this should be done alongside a PCR using flanking oligonucleotide primers to allow unbiased Sanger sequencing of the amplicon(s) to confirm it to be a *bona fide* circRNA. RT‐PCR is a straightforward and cost‐effective method; however, this approach is susceptible to false negatives, due to extensive secondary structure arresting reverse transcription, and false positives, which can arise from linear RNA contamination or rolling circle amplification (RCA) artifacts [19, 38]. Linear RNA contamination occurs when the circRNA primers also bind to sequences in linear RNA, leading to the unintended amplification of these nontarget products. To avoid this, using RNase R to eliminate linear forms prior to reverse transcription is crucial. RCA artifacts can occur during the reverse transcription process, particularly with small circRNAs, where the reverse transcriptase may inadvertently create concatemeric cDNA—essentially long chains of repeated sequences derived from a single circRNA molecule. This concatenation can significantly inflate the apparent abundance of circRNAs during PCR amplification when utilizing short‐read sequencing, leading to misleading results that overestimate the true expression levels. Employing SynCRS across a range of known sizes, where smaller circRNAs are known to be overestimated compared with larger circRNAs, can account for this effect [37, 39].

It is important to note that while RT‐PCR can be used to detect circRNAs, it is not quantitatively accurate. For quantitative analyses, qRT‐PCR is preferred due to its higher sensitivity and quantification capabilities.

### Quantitative real‐time RT‐PCR (qRT‐PCR)

Building on the principle of RT‐PCR, quantitative reverse transcriptase PCR (qRT‐PCR) enables real‐time monitoring of the amplification process, allowing for precise quantification of circRNAs (Fig. 3B). The key advantage of qRT‐PCR is its high sensitivity and wide dynamic range, making it capable of detecting low‐abundance circRNAs. Moreover, short amplicons are typically preferred in qRT‐PCR for increasing amplification efficiency and improving the sensitivity of the quantification process. However, depending on the strategy for oligonucleotide design, qRT‐PCR may struggle to distinguish between different isoforms of circRNAs that share the same BSJ, even if utilizing a probe‐based assay method. Therefore, it is essential to verify results with gel electrophoresis, as well as to add normalization strategies to ensure accurate quantification. To ensure reproducibility and consistency, the MIQE guidelines should be followed [40]. These guidelines emphasize robust design of experimental conditions, such as primer and probe design, amplification efficiency, and the inclusion of appropriate controls (e.g., nontemplate controls or housekeeping genes). Furthermore, data analysis methods, including threshold determination and normalization techniques, should be explicitly described to ensure reliable interpretation of qRT‐PCR results, particularly when quantifying low‐abundance circRNAs.

### Droplet digital PCR

For even greater sensitivity, reverse transcription droplet digital PCR (ddPCR) offers a powerful alternative to traditional PCR methods for detecting circRNAs (Fig. 3C) [41, 42]. One of the key features of ddPCR is its ability to partition the sample into thousands of individual droplets, creating microenvironments where PCR amplification occurs independently in each droplet. Following amplification, the droplets are categorized as positive or negative based on fluorescence detection, and the count of positive droplets is utilized to estimate the concentration of the target circRNA in the original samples. The distribution of positive droplets is modeled using Poisson statistics, which facilitates the calculation of confidence intervals for estimated concentrations, enhancing the reliability of results, particularly when analyzing low‐abundance targets [43]. However, the need for specialized equipment, expertise for data analysis and higher cost compared with qRT‐PCR restrict its widespread use.

### Rolling cycle amplification‐based circRNA detection

Rolling circle amplification (RCA) is an effective technique for detecting circRNA (Fig. 3D). The process starts with isolating intact total RNA from the sample and designing a specific complementary DNA primer that targets the BSJ of the circRNA [44]. This primer facilitates the conversion of circRNA into a DNA template using reverse transcriptase. RCA is then initiated, where DNA polymerase extends from the primer to generate long, repeating DNA strands complementary to the circRNA. A molecular beacon is incorporated for detection, which hybridizes to the amplified DNA and release a fluorescent signal, allowing for the quantification of circRNA. This method offers advantages over traditional qRT‐PCR, including simpler procedures and reduced costs. However, designing of specific primers and optimizing reaction conditions can be time‐consuming.

Integrating linear DNA nanostructures (LDN) into RCA can further enhance detection capabilities [45]. This approach involves generating a long DNA scaffold from a circRNA through RCA, which contains multiple binding sites for two hairpin probes, H1 and H2. When the target circRNA hybridizes with H1 probe, it triggers a conformational change that allows H2 to emit a fluorescent signal, facilitating signal amplification. The use of DNA nanostructures allows for the potential development of multiplexed assays, enabling the simultaneous detection of multiple circRNAs within a single sample. However, the method's complexity and time‐consuming nature arise from the necessity to optimize probe penetration and carefully design the distances between the probes. These factors can impact the overall effectiveness and efficiency of the assay.

### SplintQuant (PBCV‐1 DNA ligase)

SplintQuant is a method designed for the sensitive and quantitative detection of circRNAs by leveraging the unique features of their stable circular structure without reverse transcription (Fig. 3E) [39]. This approach employs a combination of molecular beacons and splint ligation techniques, making it particularly effective for identifying circRNAs. The method involves the design of specific molecular beacon probes that hybridize with the target circRNA, commonly at the BSJ. With the two oligonucleotide splints docked on the target circRNA and immediately adjacent, PBCV‐1 DNA ligase fuses these oligos. As the splint contains universal oligonucleotide overhangs, the ligation product is a perfect template for qRT‐PCR to enumerate these circRNAs. The main advantage of this approach is it does not require reverse transcription, which has been shown to bias amplification of smaller circular RNAs [39]. Other advantages of SplintQuant is high sensitivity, allowing for the detection of lowly abundance circRNAs and high specificity, discriminating even single nucleotide polymorphisms. SplintQuant is relatively rapid and straightforward, producing timely results. However, the success of this method heavily relies on the design and specificity of the constructs used, which can present challenges when dealing with novel circRNAs. Additionally, its multiplexing capabilities may be limited compared with other high‐throughput methods.

### Hybridization‐based detection

Hybridization‐based methods for detecting circRNAs employ specific nucleic acid probes that bind to the target circRNA through complementary base pairing, typically at the BSJ to enhance specificity and minimize nonspecific hybridization [46]. Various techniques utilize this hybridization principle, including northern blotting, probe‐based qRT‐PCR, microarray analysis, and RNA fluorescence *in situ* hybridization (RNA‐FISH) (Fig. 3).

### Northern blotting

Northern blotting is a classical method involving size‐dependent RNA separation via gel electrophoresis, followed by RNA transfer to a charged membrane (Fig. 3F). Detection involves RNA, DNA, or oligonucleotide probes that are complementary to the unique circRNA BSJ, enabling precise identification of circRNAs even in complex RNA mixtures. By employing probes targeting the body of the circRNA, this can distinguish circRNAs from cognate linear RNAs. Additionally, Northern blotting provides size information on the target circRNAs, which is beneficial in identifying different isoforms [47]. However, this technique is time‐consuming and labor‐intensive, involving multiple steps from RNA extraction to visualization, which can take several days to complete [47]. This lengthy process makes it less suitable for high‐throughput analysis, especially in studies requiring the examination of numerous circRNAs. Meanwhile, even for abundant circRNAs, a large quantity of high‐quality RNA may still be required. Sensitivity is another concern, while Northern blotting can effectively detect moderately abundant circRNAs, it may not be sensitive enough for lowly abundant circRNAs without transgenic overexpression.

### Microarray

Microarray analysis enables high‐throughput detection and quantification of multiple circRNAs simultaneously (Fig. 3G) [48]. In this method, RNA samples are labeled with fluorescent tags and hybridized to a microarray containing a grid of probes designed to bind to specific circRNA sequences. This approach can efficiently profile circRNA expression across various samples [49]. The primary advantage of microarray analysis is its ability to provide comprehensive data on circRNA expression patterns in a single experiment, facilitating comparative studies. However, the sensitivity of microarrays can vary, cross‐hybridization may occur if probes are not optimally designed, potentially leading to false positives. It has for instance been shown that the majority of high‐abundance circRNAs in colon cancer detected by microarray could not be validated with Nanostring nCounter technology [50]. Other limitations of microarray technology are that they can only detect circRNAs, which are previously annotated or predicted and cannot distinguish between circRNA variants sharing the same BSJ. Furthermore, microarrays generally have more limited support for various species compared with RNA‐seq approaches, which can detect circRNAs in a broader range of organisms using similar pipelines and without the need for prior knowledge of the BSJs.

### NanoString nCounter technology

Nanostring technology utilizes a unique set of probes that bind to specific target sequences, allowing for the simultaneous detection of multiple RNAs, including circRNAs, in a single sample directly on the native RNA (Fig. 3H) [51]. This is particularly beneficial in studies where understanding the expression of multiple transcripts is crucial, such as comparative gene expression analysis. Additionally, Nanostring does not require amplification or reverse transcription of RNA, which minimizes biases that can arise from these techniques, preserving the integrity of the original RNA content. However, the cost associated with the reagents and specialized equipment can be a limitation. Moreover, the design of specific probes for novel circRNAs can be time‐consuming and may face limitations in flexibility.

### Spatial transcriptomics

While bulk tissue expression analysis of circRNAs is informative, spatial transcriptomics can localize expression of transcripts to specific cells, or compartments within heterogeneous tissue architecture providing another dimension in correlating expression with biological function (Fig. 3I). Currently, only a single publication has reported spatial profiling of circRNAs with laser capture microdissection/qRT‐PCR and spatial transcriptomics using the Nanostrong GeoMx platform employing a circRNA‐specific probe [50]. This report demonstrated that expression of a panel of circRNAs was inversely correlated with cellular proliferation rates, with extratumoral cells confounding circRNA profiling in bulk tissue analysis. Additional platforms, including the Visium (10× Genomics), which utilizes oligonucleotide probes targeting the BSJ to capture RNA from a tissue section (fixed‐frozen or paraffin embedded) followed by sequencing could more affordably increase the breadth of circRNAs profiled (Fig. 3I). Performing targeted circRNA detection, alongside the default genome‐wide sequencing probes would provide cellular identity information to deconvolute circRNA expression patterns in even highly heterogeneous tissues.

### RNA fluorescence *in situ* hybridization (RNA‐FISH)

RNA‐FISH is a powerful technique that allows for the localization and visualization of circRNA within tissue sections or cell samples (Fig. 3J) [52]. CircRNAs are known for their high stability, which allows them to accumulate in cells even under conditions where linear RNAs may degrade more quickly. However, it is important to note that circRNAs exhibit slow biogenesis, meaning they tend to accumulate more prominently in quiescent cells, such as neurons and muscle cells, where cellular activity is relatively low, while circRNAs often do not reach steady‐state levels in rapidly proliferating cells due to the faster turnover of RNA in these actively dividing cells [50, 53]. RNA‐FISH is a valuable tool for visualizing and quantifying circRNAs in specific cellular contexts, providing insight into their distribution and role in gene regulation under different physiological conditions.

This method employs fluorescent or chromogenic probes that specifically hybridize to target circRNA sequences. Once hybridized, the probes can be detected using microscopy, providing spatial context regarding where the circRNA is expressed within the biological samples. Employing single‐molecule FISH (smFISH) allows detection and enumeration of individual RNA molecules and has been employed on a range of circRNAs [46, 54]. However, due to the need for multiple probes targeting sequences beyond the BSJ or other circRNA variant‐specific sequences, standard smFISH can face challenges in distinguishing circRNAs from linear RNAs as they share identical exon sequences. RNA‐FISH is particularly advantageous for studying the cellular and tissue‐specific roles of circRNAs, as it facilitates co‐localization of the circRNA with its putative target (protein, RNA, DNA or organelle). A more recent adaptation of FISH, called circFISH, allows simultaneous detection of circRNA and its cognate linear RNA to better differentiate the circRNA target [55]. FISH methods can be time‐consuming and requires meticulous optimization of probe design and hybridization conditions to ensure specificity and minimize background noise [52].

BaseScope is another advanced hybridization‐based assay that excels in detecting and visualizing RNA transcripts, including circRNAs [56]. This technique utilizes specially designed probes that target unique sequences within circRNAs, enabling high specificity in distinguishing between circRNAs and linear RNAs. One of the standout features of BaseScope is its ability to visualize RNA expression at a single‐molecule resolution. BaseScope also presents certain challenges. The complexity of the assay requires careful optimizing and validation of probes, making it more labor‐intensive compared with simpler methods. Additionally, the cost associated with BaseScope, including reagents and equipment, can be high, similar to Nanostring technology.

Additional techniques capable of addressing certain limitations of existing technologies have been proposed include RNA aptamers, multiply labeled tetravalent RNA imaging probes (MTRIPs) and molecular beacons. These have been covered elsewhere [46].

## Protein/enzyme‐based methods to detect circRNAs

### Loop‐mediated isothermal amplification

Loop‐mediated isothermal amplification (LAMP) is a rapid and specific method for detecting circRNAs, which operates at a constant temperature, typically between 60 °C and 65 °C, for amplification of target RNA (Fig. 3K) [57]. The process begins with the design of a unique set of four to six oligonucleotides that target specific regions around BSJ of the circRNA. This targeting is crucial as it enhances specificity, reducing the likelihood of amplifying linear RNAs. During the reaction, a strand‐displacing DNA polymerase synthesizes new DNA strands while displacing previously synthesized ones, leading to exponential amplification of the target circRNAs [46]. The amplification in LAMP is efficient and generates significant quantities of target circRNAs within 30–60 min. Detection of the amplified products can be achieved through various methods, including real‐time fluorescence using intercalating dyes such as SYBR Green, colorimetric detection, or gel electrophoresis. LAMP is characterized by its speed, simplicity and ability to provide quick results. However, designing effective primers can be complex, particularly when targeting a diverse range of circRNAs. Additionally, while LAMP is generally specific, there is still a risk of nonspecific amplification if the primers are not carefully optimized.

### Duplex‐specific nuclease

Duplex‐specific nuclease (DSN) assays represent another powerful enzyme‐based approach for detection of circRNAs, leveraging the specificity of DSN, which selectively degrades double‐strand DNA and RNA hybrids (Fig. 3L) [58]. The process begins with the design of molecular beacon probes that specifically hybridize to the unique BSJs of circRNAs. These probes contain a fluorophore‐quencher pair that allows for fluorescence to be suppressed until the probe is cleaved, making it an effective tool for quantifying circRNA levels [59]. After the probes have hybridized with their target circRNAs, DSN is introduced to the reaction, this enzyme degrades DNA strand of any hybrid formed, releasing both the circRNA and the fluorescently labeled probe fragment. This cyclical process enhances the sensitivity of detection by allowing the released circRNA to hybridize with additional probes, resulting in a robust amplification of the fluorescent signal. The high sensitivity of DSN assays makes them particularly effective for detecting lowly abundant circRNAs. However, the design of effective molecular beacons and optimization assay conditions can be complex, requiring expertise and careful planning. While DSN enhances sensitivity, its amplification efficiency may not match that of traditional methods such as PCR. Additionally, the success of DSN assays heavily depends on the quality and specificity of probes used, which can vary based on sample conditions and may affect overall assay performance.

### Deactivated CRISPR‐Cas

CRISPR technology to detect circRNAs represents a novel approach that leverages the specificity and efficiency of CRISPR system for RNA detection (Fig. 3M). The two primary CRISPR systems employed for this purpose are nuclease‐inactive, or deactivated Cas9 (dCas9) and dCas13 which are fused to fluorescent proteins [60, 61]. As with gene‐editing approaches, this approach requires the specific design of a single guide RNA (sgRNA) that is reverse complementary to the unique BSJ of the target circRNA. The dCas‐guide RNA ribonucleoprotein complex then binds to the circRNA which can be microscopically imaged, even in living cells [61]. Although shown for various mRNAs, this has yet to be performed for circRNAs. The applicability of this method for circRNA detection, however, may be hampered by the availability of protospacer‐adjacent motif (PAM) sequences and the potential for off‐target effects, which could limit the specificity and efficiency of circRNA detection [62].

## Concluding remarks

This brief review summarizes the strategies for researchers to detect circular RNA variants, with a summary of emerging and potential future technologies. While both the specific scientific question and access to reagents and hardware may dictate the method employed, only once the entire circRNA variant sequence has been defined, are researchers equipped to determine the most appropriate method for their detection, quantification or localisation. Combining specific enrichment/detection/amplification approaches compatible with long‐read sequencing and bespoke bioinformatics pipelines offers the most robust approach to investigate circRNA variants. With increasing publications highlighting the important functional roles of circRNAs in human health, further method development is necessary to comprehensively profile circRNA sequence variants.

## Conflict of interest

The authors declare no conflict of interest.

## Author contributions

HL, VMC, and SJC have conceptualized, written and edited the paper. All authors have read and agreed to the published version of the manuscript.
